# Further Evidence for Staphylococcal Food Poisoning Outbreaks Caused by *egc*-Encoded Enterotoxins

**DOI:** 10.3390/toxins7030997

**Published:** 2015-03-20

**Authors:** Sophia Johler, Petra Giannini, Marco Jermini, Jörg Hummerjohann, Andreas Baumgartner, Roger Stephan

**Affiliations:** 1 Institute for Food Safety and Hygiene, Vetsuisse Faculty University of Zurich, Winterthurerstrasse 272, 8057 Zurich, Switzerland; E-Mail: ils@fsafety.uzh.ch; 2 Cantonal Laboratory, Via Mirasole 22, 6500 Bellinzona, Switzerland; E-Mails: petra.giannini@ti.ch (P.G.); dss-lc@ti.ch (M.J.); 3 Agroscope, Institute for Food Sciences, Schwarzenburgerstrasse 161, 3003 Bern, Switzerland; E-Mail: joerg.hummerjohann@agroscope.admin.ch; 4 Federal Food Safety and Veterinary Office, Schwarzenburgerstrasse 155, 3003 Bern, Switzerland; E-Mail: andreas.baumgartner@blv.admin.ch

**Keywords:** staphylococcal food poisoning, enterotoxin, *seg*, *sei*, outbreak investigation, *Staphylococcus aureus*

## Abstract

Staphylococcal food poisoning represents the most prevalent foodborne intoxication worldwide. It is caused by oral intake of enterotoxins preformed by *Staphylococcus aureus* in food. The relevance of newly described enterotoxins in outbreaks of staphylococcal food poisoning is controversially discussed. Although the staphylococcal enterotoxins SEG, SEI, SEM, SEN, and SEO elicit emesis in a monkey feeding assay, there has been no conclusive proof of their emetic activity in humans. In this study, we provide further evidence suggesting that one of these enterotoxins or a combination of SEG, SEI, SEM, SEN, and SEO cause staphylococcal food poisoning. We investigated two outbreaks registered with the Swiss Federal Office of Public Health, in which only *Staphylococcus aureus* strains harboring the *egc* cluster, including *seg*, *sei*, *sem*, *sen*, and *seo* linked to typical signs of staphylococcal food poisoning were isolated. The outbreaks were caused by consumption of raw goat cheese and semi-hard goat cheese, and were linked to strains assigned to CC45 (*agr* type I) and CC9 (*agr* type II), respectively. These outbreaks provide further evidence that newly-described staphylococcal enterotoxins are likely to cause staphylococcal food poisoning in humans.

## 1. Introduction

The CDC (Centers for Disease Control) estimates that 240,000 cases of Staphylococcal Food Poisoning (SFP) occur each year in the US, leading to hopitalization in 1000 cases and to six deaths [1]. In the EU, the number of SFP outbreaks is rising, with 386 SFP outbreaks reported in 2013 [2]. The causative agents are staphylococcal enterotoxins (SEs) preformed by *Staphylococcus (S.) aureus* in food. Within two to six hours after ingestion of food containing SEs, patients present with symptoms of acute gastroenteritis, including violent vomiting and diarrhea [3]. As SFP is typically self-limiting, with only 10% of patients [4] seeking medical care, there are only a few SFP outbreaks, in which a causative *S. aureus* strain and its respective enterotoxins were unambiguously identified. 

A variety of SEs and SE-like superantigens has been described. All classical SEs (SEA-SEE) and, to a lesser degree, also most of the newly described SEs, including SEG and SEI, can elicit an emetic response in a monkey feeding assay [5,6]. However, it was shown that strains harboring *seg* and *sei* only produce very low levels of SEG and SEI [7] and only weak emetic activity was demonstrated for SEI [8]. Thus, only for the classical SEs [9] and SEH [10,11,12], there is evidence demonstrating emetic activity in humans. Still, in recent years, there have been a growing number of studies indicating that SEG and SEI may be responsible for cases of SFP in humans [13,14]. In the context of large studies characterizing a multitude of *S. aureus* strains from various outbreaks, one outbreak caused by an isolate harboring *seg*, and two outbreaks caused by isolates harboring *seg* and *sei* have been mentioned [13,14,15]. However, as no information on the outbreak investigations was provided, it is impossible to evaluate whether sufficient evidence was present to unambiguously link the strains as the cause of the SFP cases. 

The discussion is further fueled by the fact that in many outbreaks strains exhibiting both classical and newly described enterotoxin genes can be detected, but only classical enterotoxins can be identified in food and feces by commercially available immunological based methods. Thus, if food and feces samples yield positive results for even very small quantities of classical enterotoxins, outbreaks will be reported to be due to these enterotoxins, even if *seg*, *sei*, *sem*, *sen*, or *seo* genes are present. If, however, food and feces samples yield negative results for classical enterotoxins, but strains harboring *seg*, *sei*, *sem*, *sen*, or *seo* were found, the outbreak may not be reported at all as investigators question the emetic activity of SEG, SEI, SEM, SEN, and SEO in humans.

In a recent study investigating clonality and genetic characteristics of *S. aureus* strains isolated from ready-to-eat foods in Switzerland [16], we identified two cases of SFP, in which only strains exhibiting the newly-described SE genes *seg*, *sei*, *sem*, *sen*, and *seo* had been detected. In addition, in September 2014, yet another SFP outbreak linked to a *S. aureus* strain exhibiting *seg*, *sei*, *sem*, *sen*, and *seo* occurred in Switzerland. In this study, we present collected evidence from two outbreak investigations, suggesting that newly-described enterotoxins can indeed cause SFP in humans. 

## 2. Results 

### 2.1. Outbreak 2007 

On 4 June 2007, two people purchased Robiola type fresh cheese made from thermized goat milk at a store in Ticino, the Italian-speaking part of Switzerland. While one of them stored the cheese at refrigeration temperatures to serve it to his family at dinner, the other gave it as a present to a friend who consumed the cheese right away. Within 1.5–3 h after consumption, all five people that had ingested the cheese exhibited signs of acute gastroenteritis, such as nausea, abdominal cramps, vomiting, and, in some cases, diarrhea (Table 1). 

**Table 1:** Outbreak due to Robiola goat cheese on 4 June 2007.

Patient	Age	Sex	Hospitalized	Clinical signs	Incubation time
Patient 1	54	M	Yes	Nausea, vomitus, abdominal cramps	1.5–2 h
Patient 2	54	F	Yes	Nausea, vomitus, abdominal cramps, diarrhea	1.5–2 h
Patient 3	23	M	Yes	Nausea, vomitus, abdominal cramps, diarrhea	1.5–2 h
Patient 4	21	M	Yes	Nausea, vomitus, abdominal cramps	1.5–2 h
Patient 5	38	M	No	Nausea, vomitus, abdominal cramps, diarrhea	2.5–3 h

Thirteen Robiola samples were taken from households of the patients, as well as from Robiola cheese produced by the manufacturer on the day after the outbreak. While the maximum level of coagulase positive *Staphylococci* (CPS) detected among samples obtained from the manufacturer was 4.6 × 10^5^ CfU/g cheese, samples obtained from cheese associated with illness exhibited between 6.7 × 10^6^ and 2.6 × 10^7^ CfU/g cheese. As all cheese samples tested negative for the classical enterotoxins SEA-SEE using Vidas SET2, three *S. aureus* strains were selected for further characterization by DNA microarray (Table 2) to test not only for genes encoding the classical enterotoxins (*sea-see*), but also for genes encoding the newly described enterotoxins (*seg*, *seh*, *sei*, *sej*, *sek*, *sel*, *sem*, *sen*, *seo*, *sep*, *seq*, *ser*, *selu*). The only enterotoxin genes detected by DNA microarray were the genes of the enterotoxin gene cluster *seg*, *sei*, *sem*, *sen*, and *seo*. Microarray profiles showed that all three isolates represented the same *S. aureus* strain and belong to clonal complex (CC) 45 and *agr* type I. Complete microarray hybridization results for all three outbreaks are provided as a supplemental file. No *ses* and *set* enterotoxin genes were detected by PCR screening.

**Table 2:** Selected Robiola goat cheese samples obtained during the 2007 outbreak from households of SFP patients. *S. aureus* isolates were characterized by DNA microarray to test for genes encoding the classical, as well as newly described enterotoxins.

ID	CPS in CfU/g	Source	SET Vidas	Classical SE genes	Newly described SE genes	Clonal complex
SFP1_1	7.1 × 10^6^	Patients 1–4	No SEA-SEE	None	*seg*, *sei*, *sem*, *sen*, *seo*	CC45
SFP1_2	7.6 × 10^6^	Patients 1–4	No SEA-SEE	None	*seg*, *sei*, *sem*, *sen*, *seo*	CC45
SFP1_3	2.6 × 10^7^	Patient 5	No SEA-SEE	None	*seg*, *sei*, *sem*, *sen*, *seo*	CC45

### 2.2. Outbreak 2014 

On 7 September 2014, an SFP outbreak due to Formagella d’alpe goat cheese occurred in the Ticino region, the Italian-speaking part of Switzerland. Five people consumed the semi-hard goat cheese made from raw milk, and fell ill within the following 6 h (average incubation time 3.8 h), exhibiting acute symptoms of gastroenteritis, such as nausea and vomiting (Table 3). 

**Table 3:** Outbreak due to consumption of a semi-hard cheese from raw goat milk (Formagella d’alpe) on 7 September 2014.

ID	Age	Sex	SFP	Incubation time	Clinical signs	Consumed food items
Person 1	80	F	Yes	6 h	Nausea, vomitus	Semi-hard cheese from raw goat milk, fresh cheese, potatoes
Person 2	50	M	Yes	3.5 h	Nausea, vomitus	Semi-hard cheese from raw goat milk, fresh cheese, potatoes
Person 3	52	F	Yes	4.5 h	Nausea, vomitus	Semi-hard cheese from raw goat milk, fresh cheese, potatoes
Person 4	48	F	Yes	2.5 h	Nausea, vomitus	Semi-hard cheese from raw goat milk
Person 5	14	M	Yes	2.5 h	Nausea, vomitus	Semi-hard cheese from raw goat milk
Person 6	42	M	No	-	None	Semi-hard cheese from raw goat milk

The investigative team performed a case control study, in which the semi-hard goat cheese was identified as the cause of the outbreak (odds ratio = 25). The cheese was a Formagella d’alpe that had been produced on 1 August and had, thus, been ripened for five weeks before consumption. The cheese tested negative for the classical enterotoxins SEA-SEE in the mini Vidas SET2. A total of 2.6 × 10^3^ CfU CPS per g cheese were detected. Single colonies consistent with a *S. aureus* phenotype on rabbit plasma fibrinogen agar were screened for genes encoding enterotoxins*.* Four different *S. aureus* strains were detected (Table 4). Three of these strains exhibited no enterotoxin genes. Only one strain (SFP3_1) exhibited the genes of the enterotoxin gene cluster *seg*, *sei*, *sem*, *sen*, and *seo* but no other enterotoxin genes including *ses* and *set*. SFP3_1 was assigned to CC9 and *agr* type II. 

**Table 4:** *S. aureus* strains isolated from the semi-hard goat cheese associated with the 2014 outbreak of SFP in Ticino. Strains were characterized by DNA microarray to test for genes encoding the classical, as well as newly described enterotoxins. No classical enterotoxins (SEA-SEE) were detected in the cheese samples by mini Vidas SET2.

ID	Clonal complex	Classical SE genes	SEA-SEE (mini Vidas SET2)	Newly described SE genes
SFP2_1	CC9	None	None	*seg*, *sei*, *sem*, *sen*, *seo*
SFP2_2	CC45	None	None	None
SFP2_3	CC130	None	None	None
SFP2_4	CC522	None	None	None

## 3. Discussion

The results of our study illustrate that risk factor analysis in SFP outbreak investigations should not be solely based on CPS counts. In the outbreak in 2007 due to Robiola fresh cheese from thermized goat milk, CPS count per g food considerably exceeded the legal limit of 10^5^ CfU/g food, which is considered a health risk (EU 2073:2005). However, in the outbreak in 2014, we detected only 2.6 × 10^3^ CfU of CPS per g cheese at the time of consumption. However, by this time, the semi-hard cheese from raw goat milk had been ripened for five weeks. In semi-hard raw milk cheese ripened at 10 °C and 92% air humidity, CPS counts were shown to peak around day seven of the ripening process and then gradually decline until they commonly fall below the detection limit at week nine of the ripening process [17]. The strains causing the SFP outbreaks in this study were assigned to CC9 and CC45. CC9 is particularly frequently found in strains isolated from caprine mastitis milk [18].

In this study, we present outbreaks, in which only strains harboring *seg* and *sei*, and other genes encoded by *egc* were detected. We omitted any SFP outbreaks, in which classical enterotoxin genes or *seh* were detected in the same or another *S. aureus* strain.

In both outbreaks described in this study, the causative strain carried *seg*, *sei*, *sem*, *sen*, *seo*, and *selu*. Currently, no data on emetic activity of SElU is available. Administering SEM, SEN, and SEO to cynomolgus monkeys at 100 μg/kg showed weak emetic activity and resulted in vomiting of one in seven, two in six, and one in eight animals, respectively [6]. ED_50_ of classical enterotoxins in monkeys ranges between 0.9 and 20 μg/kg [19,20]. In rhesus monkeys, SEG and SEI provoke diarrhea or pronounced lethargy in all tested animals and emesis in four out of six (80 μg/kg SEG) and one out of four (150 μg/kg SEI) animals [8]. While the emetic activity of SEI in the rhesus monkey seems rather low, it provokes vomiting in the house musk shrew at an emetic activity comparable to SEA and far exceeding SEB, SEC, and SED [21]. 

Taking into consideration data from feeding assays, as well as reports of strains carrying *seg* and *sei* in association with intoxications [13,14,15], it is most likely that the SFP outbreaks described in this study were caused by SEG and/ or SEI. It is also highly likely that the semi-hard goat cheese that caused the SFP outbreak exhibited far higher CPS counts earlier in the ripening process and that during this phase large amounts of enterotoxins were produced by *S. aureus*. Even when the number of viable cells of the organism is reduced, the extremely stable enterotoxins remain emetically active and can cause SFP [22]. 

Interestingly, both presumptive SEG and SEI associated SFP outbreaks described in this study are linked to goat milk products. This is consistent with previous studies reporting that the enterotoxin gene combinations *sec/seg/sei* and *seg*/*sei* were frequently detected among *S. aureus* strains isolated from cheese made of raw goats’ milk [23]. Interestingly, both SFP outbreaks were linked to goat milk products. As there are currently no suitable methods for detection of SEG and SEI or other newly-described enterotoxins in food and feces, further research will be necessary to determine the individual role of these enterotoxins in SFP outbreaks. 

## 4. Materials and Methods 

### 4.1. Screening for Staphylococcal Enterotoxins 

Vidas SET and mini Vidas SET2 (bioMérieux, Marcy d’Etoile, France) were used to screen for the classical enterotoxins SEA-SEE in food. Cheese extracts were prepared and processed following the manufacturer’s instructions. 

### 4.2. Bacterial Isolation and Identification

Columbia agar with 5% sheep blood (bioMérieux, Marcy d’Etoile, France) and Baird Parker Rabbit Plasma Fibrinogen (RPF, Oxoid, Basel, Switzerland) agar were used to screen for *S. aureus.* The number of coagulase-positive *Staphylococci* per g food was determined using the plate count technique on RPF and following the EN ISO 6888-2 protocol. Several presumptive *S. aureus* colonies were selected from RPF agar and were further characterized. 

### 4.3. Microarray-Based Genotyping

The *S. aureus* Genotyping Kit 2.0 ArrayStrip platform (Alere Technologies GmbH, Jena, Germany) was used for DNA microarray profiling according to the manufacturer’s instructions. DNA microarray determines the presence/absence of over 300 allelic variants of resistance and virulence genes including genes encoding classical (*sea-see*), and newly-described enterotoxins and enterotoxin-like superantigens (*seg*, *seh*, *sei*, *sej*, *sek*, *sel*, *sem*, *sen*, *seo*, *sep*, *seq*, *ser*, *selu*) [24]. It also allows for *S. aureus* species confirmation and predicts assignment of the tested isolates to clonal complexes, as well as *agr* types [24]. The microarray has been used to detect enterotoxin genes in *S. aureus* linked to outbreaks [25,26] or isolated from different foods [16,27,28].

### 4.4. PCR Screening for ses and set

As the DNA microarray does not include probes targeting *ses* and *set*, screening for these enterotoxin genes was performed using a PCR approach, a GoTqy PCR system (Promega AG, Dübendorf, Switzerland) and primers are published elsewhere [29].

### 4.5. Odds Ratios

In the outbreak in Ticino in 2014, family members, as well as persons that had consumed semi-hard cheese from a different batch from the same producer were used as controls (*n* = 6). Information on food ingested up to 48 h before the outbreak was collected from all cases and controls. As all SFP patients had ingested the suspicious cheese, zero cases reported no exposure to the cheese (*c* = 0), and *c* was replaced by 1 to allow for OR calculation.

## Supplementary Materials

Supplementary materials can be accessed at: http://www.mdpi.com/2072-6651/7/3/0997/s1.
